# Combining 5G New Radio, Wi-Fi, and LiFi for Industry 4.0: Performance Evaluation

**DOI:** 10.3390/s24186022

**Published:** 2024-09-18

**Authors:** Jorge Navarro-Ortiz, Juan J. Ramos-Munoz, Felix Delgado-Ferro, Ferran Canellas, Daniel Camps-Mur, Amin Emami, Hamid Falaki

**Affiliations:** 1 Department of Signal Theory, Telematics and Communications, University of Granada, 18014 Granada, Spain; jjramos@ugr.es (J.J.R.-M.); felixdelgado@ugr.es (F.D.-F.); 2 Research Center on Information and Communication Technologies, University of Granada, 18014 Granada, Spain; 3 i2CAT Foundation, 08034 Barcelona, Spain; ferran.canellas@i2cat.net (F.C.); daniel.camps@i2cat.net (D.C.-M.); 4 Smart Internet Laboratory-HPN, Department of Electrical and Electronics Engineering (EEE), University of Bristol, Bristol BS8 1UB, UK; amin.emami@bristol.ac.uk (A.E.); hamid.falaki@bristol.ac.uk (H.F.)

**Keywords:** 5G, LiFi, Wi-Fi, MPTCP, multi-connectivity, scheduling

## Abstract

Fifth-generation mobile networks (5G) are designed to support enhanced Mobile Broadband, Ultra-Reliable Low-Latency Communications, and massive Machine-Type Communications. To meet these diverse needs, 5G uses technologies like network softwarization, network slicing, and artificial intelligence. Multi-connectivity is crucial for boosting mobile device performance by using different Wireless Access Technologies (WATs) simultaneously, enhancing throughput, reducing latency, and improving reliability. This paper presents a multi-connectivity testbed from the 5G-CLARITY project for performance evaluation. MultiPath TCP (MPTCP) was employed to enable mobile devices to send data through various WATs simultaneously. A new MPTCP scheduler was developed, allowing operators to better control traffic distribution across different technologies and maximize aggregated throughput. Our proposal mitigates the impact of limitations on one path affecting others, avoiding the Head-of-Line blocking problem. Performance was tested with real equipment using 5GNR, Wi-Fi, and LiFi —complementary WATs in the 5G-CLARITY project—in both static and dynamic scenarios. The results demonstrate that the proposed scheduler can manage the traffic distribution across different WATs and achieve the combined capacities of these technologies, approximately 1.4 Gbps in our tests, outperforming the other MPTCP schedulers. Recovery times after interruptions, such as coverage loss in one technology, were also measured, with values ranging from 400 to 500 ms.

## 1. Introduction

The fifth-generation mobile initiative, 5G, is a tremendous and collective effort to specify, standardize, design, manufacture, and deploy the next cellular network generation. Indeed, 5G is set to revolutionize connectivity across numerous private sectors, driving digital transformation and enabling advanced use cases that cannot be supported by current wireless access technologies (WATs). The potential impact is underscored by projections that the 5G-enabled market in key vertical industries could reach USD 700 billion by 2030, with a compound annual growth rate of 50 percent between 2020 and 2026 [1]. Among the sectors likely to benefit from 5G’s capabilities, tourism, healthcare, retail, transport hubs, sports facilities, and manufacturing, all of which require diverse and stringent wireless access needs that only 5G can currently fulfill, can be included.

In the context of Industry 4.0, a significant advantage of adopting 5G in factories is the reduction in expensive and cumbersome cabling, which currently limits device connection density and hinders the mobility of both people and machines [2,3]. Industrial 5G Non-Public Networks (NPNs) demand strict performance standards, such as throughput of 500 Mb/s per device, high connection density of 100 devices per square meter, and service reliability of eight 9s, as outlined in 3GPP TS 22.104. Meeting these demands requires the integration of various technologies to enhance wireless resources in dense user environments, ensuring high data rates and supporting redundant transmission for reliability.

To address these demands, an integrated 5G New Radio (5G NR) and Wireless Fidelity (Wi-Fi) network can be further strengthened by incorporating another non-3GPP technology: light fidelity (LiFi) [4]. LiFi offers high indoor connection density through its narrow light beams and enhanced security due to the localized nature of light confinement. Thus, non-3GPP technologies like Wi-Fi and LiFi complement 5G NR, enabling the network to meet the stringent requirements outlined above.

In this paper, we present a novel multi-connectivity scheduler implemented for the 5G-CLARITY project [5]. This project belongs to the third phase of the European 5G-PPP (5G Infrastructure Public Private Partnership) initiative [6]. Its main objective is to investigate how the concept of private 5G networks should evolve beyond 3GPP (3rd Generation Partnership Project) Release 16. One of the main pilars of this project is the development of novel user and control plane components that will integrate different radio access technologies to enhance 5GNR (5G New Radio) capabilities in terms of peak data rate, latency and reliability.

To achieve this goal, 5G-CLARITY relies on the N3IWF (Non-3GPP InterWorking Function) to establish a direct connection between non-3GPP Wireless Access Technologiess (WATs) and the 5G core. To enable the UE to simultaneously use 3GPP and non-3GPP WATs, 5G-CLARITY adopts the AT3S framework defined in 3GPP R16 [7], using MPTCP (Multi-Path TCP) [8] as the AT3S user plane function. MPTCP enables the parallel use of several IP addresses/interfaces by modifying the TCP (Transmission Control Protocol) layer in the kernel of the corresponding operating system.

Adopting an O-RAN (Open Radio Access Network) architecture in the RAN (Radio Access Network), 5G-CLARITY features an RAN intelligent controller (RIC) that can gather near-real-time telemetry from the different WATs. The RIC can then host an xApp that controls the amount of traffic transmitted through each component WAT for a given PDU session. Thus, a key innovation of this framework is to permit the development of external network functions, such as xApps deployed in the 5G-CLARITY RAN cluster, to control the proportion of traffic transmitted by each UE (User Equipment) device through each interface based on near-real-time access network telemetry.

5GNR, Wi-Fi, and Light Fidelity (LiFi) are complementary technologies. On the one hand, 5G NR allows for excellent performance with extensive coverage thanks to the use of licensed frequency bands, which avoids interference from other systems. On the other hand, Wi-Fi offers very high throughput, but its performance heavily relies on the location of access points and potential interferences from other Wi-Fi networks or other systems. Lastly, LiFi has a lower data transfer rate than other technologies, although it is expected to reach rates close to 10 Gbps with the recent approval of the 802.11bb standard [9]. Additionally, it does not suffer from the security issues inherent in Wi-Fi due to the properties of LiFi. Due to these characteristics, 5G NR, Wi-Fi, and LiFi were employed in the 5G-CLARITY project for industrial and tourism use cases.

Given the wide disparity in available bandwidth and latencies provided by 5GNR, Wi-Fi, and LiFi, another key component of the 5G-CLARITY multi-connectivity framework is a weighted MPTCP scheduler that controls the amount of traffic transmitted through each WAT. Furthermore, as it will be seen in the performance evaluation, this scheduler will allow for maximizing the aggregate throughput transmitted by the terminal, unlike other schedulers in the literature. This approach was validated in a real production environment provided by BOSCH in Aranjuez (Madrid, Spain) [10], where some inefficiencies were observed that have been solved using the scheduler presented in this paper.

The rest of the article is organized as follows. Section 2 outlines the key enabling technologies in 5G that support the envisioned use cases: enhanced Mobile BroadBand (eMBB), Ultra-Reliable and Low Latency Communications (URLLC), and massive Machine-Type Communications (mMTC). Section 3 describes the operation of MPTCP. Section 4 summarizes the works in the literature related to MPTCP in wireless and mobile networks. Section 5 describes our multi-connectivity scheduler. The performance evaluation is analyzed in Section 6. Finally, the main conclusions are drawn in Section 7.

## 2. Fifth Generation-Enabling Technologies

Fifth-generation technology is designed to support three primary use cases: enhanced Mobile Broadband (eMBB), Ultra-Reliable Low-Latency Communications (URLLC), and massive Machine-Type Communications (mMTC). Each of these use cases is supported by a set of key technologies [11,12,13,14], which are described next.

### 2.1. Enhanced Mobile BroadBand (eMBB)

eMBB focuses on providing higher data rates, improved capacity, and better user experiences for applications like HD video streaming, VR/AR, and cloud-based services. The main key technologies to support eMBB are summarized hereafter.

- Millimeter Wave (mmWave) Spectrum. mmWave utilizes higher frequency bands (24 GHz and above) to provide wider bandwidths which enable multi-gigabit-per-second data rates.
- Massive MIMO (Multiple Input Multiple Output). It employs a large number of antennas at the base station to improve spectral efficiency and increase network capacity.
- Carrier Aggregation. It combines multiple frequency bands to increase the effective bandwidth, thereby enhancing data rates.
- Beamforming. It directs signal beams toward specific users, improving signal quality and extending coverage, particularly in dense urban environments.

### 2.2. Ultra-Reliable Low-Latency Communications

URLLC is designed for applications requiring extremely low latency and high reliability, such as autonomous vehicles, industrial automation, and remote surgery. The main key technologies to support URLLC are summarized next.

- Network slicing (NS). NS creates virtualized and independent logical networks on the same physical infrastructure, allowing tailored quality of service (QoS) for specific applications with stringent requirements.
- Edge Computing (MEC, Multi-access Edge Computing). MEC brings computing resources closer to the end-user to reduce latency by processing data near the point of generation.
- Flexible Frame Structure. It introduces mini-slots and other flexible frame configurations to reduce transmission time intervals, thereby enabling lower latency.
- Fifth-generation New Radio air interface. It offers new transmission techniques and scheduling mechanisms optimized for low-latency communication.

### 2.3. Massive Machine-Type Communications

mMTC is aimed at supporting a vast number of IoT devices with low data rates but requires extensive connectivity. The main key technologies to support mMTC are summarized next.

- Narrowband IoT (NB-IoT) and LTE-M. Specialized technologies within 5G are used to support low-power, low-cost, and long-range connectivity for IoT devices.
- Device density management. Techniques such as grant-free access and efficient signaling are used to manage the massive number of connected devices without overwhelming the network.
- Energy-efficient protocols. They focus on minimizing power consumption for IoT devices, enabling batteries to live longer even in massive deployment scenarios.

### 2.4. Cross-Cutting Technologies

The following technologies collectively enable 5G to meet the diverse requirements of enhanced Mobile Broadband, Ultra-Reliable Low-Latency Communications, and massive Machine-Type Communications, paving the way for a wide range of new applications and services.

- Software-Defined Networking and Network Function Virtualization. They permit flexible and efficient network management, dynamic resource allocation, and network slicing to support the diverse requirements of eMBB, URLLC, and mMTC.
- AI and Machine Learning. They are used for network optimization, predictive maintenance, and dynamic resource management across all use cases.
- Multi-connectivity. The integration of multiple WATs with diverse characteristics, such as 5GNR, Wi-Fi, and others, facilitates more effective fulfillment of the varied requirements of 5G use cases.

## 3. MPTCP Overview

Standard TCP connections are identified by a four-tuple, which includes the source and destination IP addresses and source and destination ports, whose packets are sent through a single link.

In the case of MPTCP, defined in RFC 6824 [8], several paths—called subflows—are aggregated to create one TCP connection. The MPTCP protocol includes operations to handle when and how to add or remove paths, to be compatible with legacy TCP hardware (e.g., firewalls may reject TCP connections if the sequence numbers are not successive) and to define a fair congestion control strategy between the different links and the different hosts.

MPTCP introduces the following new mechanisms. First, the subflow system, used to gather multiple standard TCP connections. Subflows are identified during the TCP three-way handshake and, after that, an application may add or remove subflows. Second, MPTCP includes a DSS (Data Sequence Signal) option which contains a data sequence number and an acknowledgment number. DSSs allow us to receive data from multiple subflows in the original order, without any corruption. Finally, a modified retransmission protocol handles congestion control and reliability.

**Figure 1:**
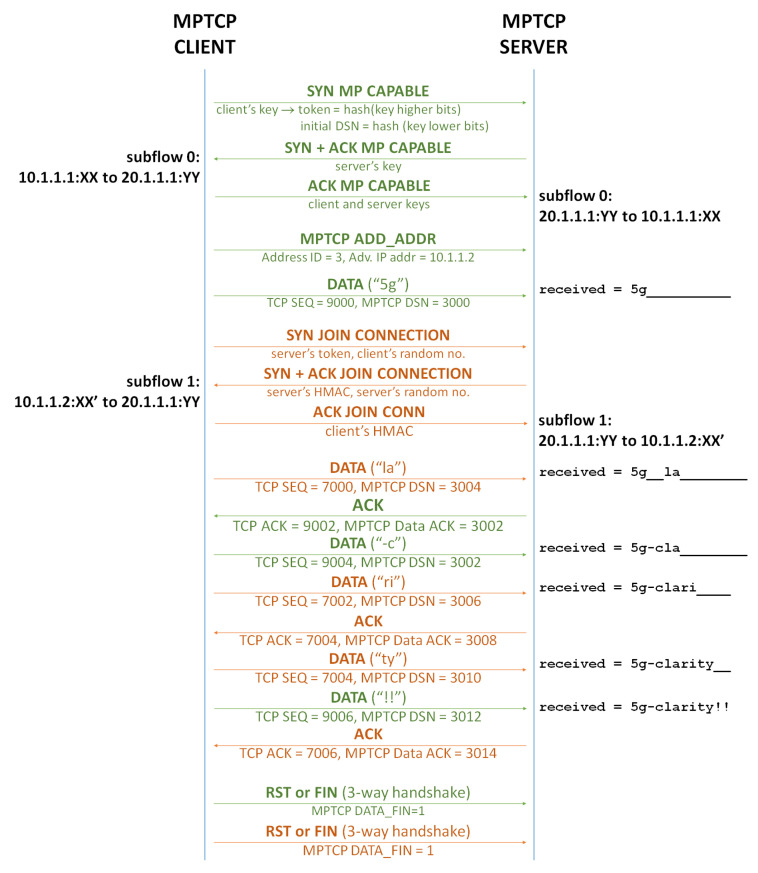
MPTCP message exchange assuming, as an example, two network interfaces on the client and one network interface on the server. Sublow 0 is shown in green, while sublow 1 is shown in orange.

An example of the MPTCP message exchange is shown in Figure 1, which includes the establishment of the different subflows (TCP SYN messages, with the MP CAPABLE option for the first subflow and the JOIN CONNECTION option for the subsequent subflows), the TCP sequence (SEQ) and TCP acknowledgment (ACK) numbers which are subflow-dependent, and the MPTCP sequence (DSN) and acknowledgment (ACK) numbers which are common for all subflows.

In this paper, we utilize the MPTCP implementation from [15] (version 0.96), which includes three main modules: the path manager, the scheduler, and the congestion control scheme.

The path manager controls the establishment of new subflows. For our experiments, we utilize the full mesh path manager, which creates all the possible subflows considering the IP addresses of the client and the server.

At the socket level, the MPTCP scheduler selects the next packet and decides which subflow will be selected for transmission. Three different schedulers are included, which are the following:

- Default. This scheduler selects the subflow with the smallest TCP *Smoothed Round Trip Time* (SRTT) among all the subflows having available space within their Congestion Windows (CWs).
- Round-Robin (RR). It transmits traffic in a Round-Robin fashion. The number of consecutive segments to be sent over each subflow can be configured (one by default). In addition, it can be specified whether the scheduler tries to fill the CW on all subflows or leaves open space in the congestion window to achieve real Round-Robin behavior.
- Redundant. Traffic will be transmitted on all available subflows with space in the CW in a redundant way, i.e., repeating the same packets through all the different paths.

In an MPTCP connection, the TCP subflows may traverse paths with varying characteristics. For instance, a subflow that is transmitted over Wi-Fi experiences a considerably lower Round Trip Time (RTT) than a subflow that is sent over 5GNR. For schedulers such as the default and the Round-Robin, the application has the capability to fill the congestion window of each subflow and, subsequently, schedules packets as soon as available space is found. This *ack-clock* effect can lead to out-of-order delivery at the receiver’s end. As MPTCP ensures in-order delivery, this can cause the packets scheduled on the low-delay subflow to be held up until the high-delay subflow’s packets arrive in the out-of-order queue of the receiver, resulting in *Head-of-Line* (HoL) blocking [16]. This HoL blocking has a high negative impact on the performance of RR when the WATs have different characteristics. For that reason, we developed a novel scheduler which is described in Section 5.

The last module, the *congestion control* mechanism, tries to compensate for the total sending rate of one MPTCP connection becoming extensively high compared to single paths’ TCP connections. This happens when the associated subflows increase their own CWs independently. To avoid this unfair situation, MPTCP congestion control schemes couple the increases in the different CWs. In particular, we use the Opportunistic Linked Increase Algorithm (OLIA) in this work.

## 4. State of the Art

Since the literature about Multi-Path TCP is abundant, in this section we will focus on the works that employ mobile and/or wireless networks.

Chen et al. [17] characterized the behavior of single-path and multi-path data transport for 3G/4G and Wi-Fi networks, evaluating major US 4G and 3G cellular networks in terms of throughput, packet loss, and RTT. The authors stated that the MPTCP connection should perform at least as well as the best single-path TCP connection of all the used paths, but not as well as the theoretical aggregation of the independent single-path TCP connections. Since this work was conducted in 2012, 4G connections achieved low throughput compared to nowadays networks, with a maximum of 15.6 Mbps and average RTTs between 39 and 170 ms. It was similar for Wi-Fi, with up to 6 Mbps and RTTs of approx. 20 ms. These high RTTs limit the throughput of the 4G connections due to the size of the TCP advertised window. Experiments show a slight increase (up to 19.6 Mbps) compared to the best single-path connection. Mobility scenarios with handovers between 3G and 4G and with intermittent Wi-Fi coverage are also successfully evaluated. Although this paper is very interesting and has some similarities with our work, there are two remarkable differences. Firstly, current mobile and wireless networks are much higher-performant, achieving several hundreds of Mbps in the case of 5G and more than 1 Gbps in the case of Wi-Fi 6. Moreover, RTTs are highly reduced (20–30 ms for 5G, 2–3 ms for Wi-Fi). And secondly, our proposed scheduler is able to achieve near the aggregated throughput of all the paths, which is much higher than that of the best single-path connection.

In another paper [18], Chen also analyzed two limitations of MPTCP when using mobile (3G/4G) and Wi-Fi networks. Due to the high RTTs in cellular networks compared to Wi-Fi, the delayed startup of additional subflows in MPTCP can limit the throughput of small data transfers. On the contrary, large data transfers may have performance degradation due to the inflated and varying RTTs of cellular subflows compared to the small and stable RTTs of Wi-Fi subflows. The authors proposed to disable the default idle restart functionality and reduce the window size of subflows with too many out-of-order packets to improve MPTCP performance. These results were in line with their work in [19] with measurements from real mobile and wireless networks. As discussed in Section 5, our proposal prevents limitations in one path from affecting other paths and avoids the *Head-of-Line* blocking problem without modifying the TCP congestion window.

Paasch et al. [20] proved the feasibility of using MPTCP for 3G/Wi-Fi vertical handover even for demanding applications, such as VoIP. They considered three modes of operation for MPTCP: full-MPTCP (all possible subflows are used), backup (all possible subflows are established and only one subflow is used), and single-path modes (only one subflow is established). They measured goodput (amount of useful data successfully transmitted over a network) and application delay. They also analyzed the impact of losing MPTCP signaling to create/remove subflows. In contrast to our work, this paper focuses on vertical handovers in slow network connections (up to 2 Mbps for 3G and 8 Mbps for ADSL-WiFi) and targets very low-speed applications (transmitting at 500 Kbps), whereas our research aims to maximize aggregate throughput with values exceeding 1.5 Gbps.

In [21], the authors proposed a two-timescale algorithm with theoretical performance guarantees that reduces energy consumption up to 22%. They analyzed which subflows should be used (path selection) and their sending rates (congestion control). The simulations assumed that devices had 3G, 4G, and Wi-Fi interfaces and that their parameters were fixed (data rate and power consumption). Since the paper is from 2014, throughput figures were low (lower than 13 Mbps for all technologies). Additionally, since the results were based on simulations, it remains unclear how the authors implemented MPTCP in their simulator and whether they accounted for realistic MPTCP performance degradations.

In [22], Mahmud et al. evaluated the performance of MPTCP for devices with 4G/5G. For this purpose, they implemented a testbed based on NS3-DCE (Network Simulator 3 with Direct Code Execution) with MPTCP Linux Kernel v0.90. The scenario included a 5G network with an NSA setup, in which the UE connects simultaneously to 4G and 5G mmWaves. This work compared the performance using different schedulers (default, Round-Robin, redundant, Early Completion First (ECF), and Block Estimation (BLEST)) and different congestion control algorithms (Linked Increase Algorithm (LIA), OLIA, Balanced Linked Increase Algorithm (BALIA), and Weighted Vegas (wVegas)). BLEST achieved the best performance since it was designed to avoid HoL blocking. Although results were interesting, they did not evaluate MPTCP operation with high throughput (i.e., higher than 1 Gbps), and they also did not propose new solutions to improve end-users’ experienced data rates. Additionally, since the performance evaluation was based on simulations, it is not clear whether realistic MPTCP performance degradations were considered.

Lan Ding et al. [23] studied the performance of TCP and MPTCP. In the case of MPTCP, they employed two mobile phones, one with 4G and another with 5G, which were connected through Wi-Fi to a laptop. Their findings show that commercial 5G faces a dilemma in maintaining in-network buffers for TCP, and due to the 4G co-locating NSA model, applying MPTCP over 5G and 4G does not necessarily improve communication reliability. They also employed crowdsourced speed test results to check the differences on 5G performance across China. The authors used the implementation from [15] (v0.95.1) with the default packet scheduler, where the data rate is determined by the path with the lowest RTT. Therefore, their work did not focus on optimizing aggregate throughput, as ours does.

In [24], the authors presented a novel MPTCP congestion control scheme named Delay-Equalized FasT (DEFT) that shows fast responsiveness for mmWaves changing from line-of-sight (LOS) to non-LOS. DEFT includes a delay-equalizing algorithm which minimizes additional reordering delay in the receive buffer. Its performance was evaluated using NS3 and compared to LIA, BALIA, and wVegas. However, the device included 5G and a wired connection to evaluate TCP friendliness. This may not be realistic for real equipment. In addition, MPTCP performance was also not compared to the aggregated throughput of both paths.

Garcia et al. [25] compared the performance of MPTCP and TCP using 5G and Wi-Fi links across eight combinations of MPTCP schedulers and congestion control algorithms available in the Linux kernel. Their findings showed that MPTCP underperformed relative to TCP in most cases, primarily due to the asymmetry between the paths. In contrast, our approach mitigates the impact of limitations on any single path, ensuring that other paths remain unaffected, while effectively aggregating the capacities of different links. This is achieved despite the notable asymmetry in throughput and latency across 5G, Wi-Fi, and LiFi.

Singh et al. [26] proposed a Smart MPTCP (S-MPTCP) path controller that optimally manages MPTCP subflows across various network interfaces. By leveraging information exchanged during connection setup, the controller maps clients to the most suitable network interface. The S-MPTCP architecture was evaluated under varying 5G mmWave channel conditions, particularly for video traffic, and dynamically routes traffic over the best available paths to optimize network performance, reducing both data and power consumption. Live air experiments and simulations confirmed that S-MPTCP delivers higher throughput, with lower 5G data utilization compared to legacy MPTCP under similar conditions. While this work is highly interesting, its focus on video traffic and Wi-Fi offloading limits its ability to fully optimize aggregate throughput.

Gentili et al. [27] utilized a Wi-Fi mesh network alongside 5G and an MPTCP scheduler to enhance 5G performance in scenarios with limited uplink capacity or unreliable coverage. They employed the Round-Robin scheduler from the Linux Kernel MPTCP implementation. Their measurements showed that reconnecting to a 5G or mesh Wi-Fi network during coverage gaps is time-consuming. These findings highlight the importance of ensuring adequate network overlap and improving the Wi-Fi mesh network, as it experienced higher packet retransmissions. Both 5G and Wi-Fi throughputs were around 50 Mbps in the tunnel, which is significantly lower compared to our setup.

Compared to these studies, our proposal not only maximizes the aggregated data rate and achieves exceptionally high throughput (exceeding 1.5 Gbps in our laboratory testbed) but also introduces a mechanism to adjust the proportion of traffic sent via each wireless technology. Moreover, to the best of our knowledge, no other works in the literature have designed or evaluated the performance of MPTCP schedulers over LiFi, 5GNR, and Wi-Fi.

## 5. MPTCP Weighted Round-Robin (WRR) Scheduler

One of the key requirements of the 5G-CLARITY project for the 3GPP AT3S load-balancing steering mode is the ability to aggregate traffic from the available WATs and control the proportion of traffic routed through each WAT [28].

For this, we propose utilizing a scheduler that implements a true Weighted Round-Robin algorithm (freely available for Linux Kernels 5.4 and 5.5 at [29]) that allows subflows to be assigned different numbers of packets per round, with weights $\omega_{subflow_{i}}\in N$. These weights can be configured using *sysctl* system calls, enabling us to (1) dynamically adjust the proportion of traffic sent through each WAT and (2) achieve maximum aggregated throughput when appropriate weights are applied. However, if the weights are not calculated correctly, it may underperform in comparison to other schedulers.

Our implementation is based on the Round-Robin scheduler described in Section 3. To mitigate the *Head-of-Line* blocking issue (discussed in Section 3), we use the *CWND_LIMITED* parameter available in the RR scheduler. When this parameter is set to “*Y*” (yes), the scheduler attempts to fill the congestion window on all subflows, thereby increasing the data rate for subflows associated with faster network interfaces. However, as mentioned beforehand, this can lead to HoL blocking, where the performance of one subflow impacts the behavior of others.

However, if *CWND_LIMITED* is set to “*N*” (no), the scheduler enqueues exactly the number of packets for each subflow based on its assigned weight, even if the congestion window is not fully utilized. This ensures that the data rate for each network interface is independent of the others, implementing a true Weighted Round-Robin policy and effectively avoiding HoL blocking.

**Figure 2:**
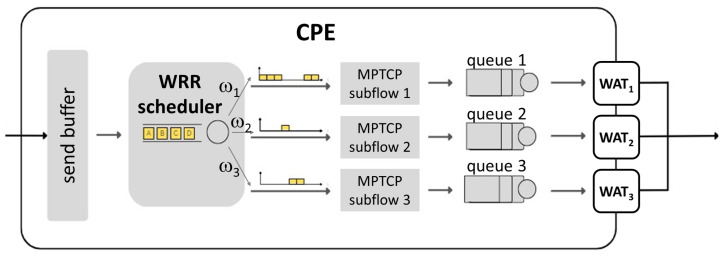
MPTCP WRR scheduler.

Figure 2 shows the operation of the WRR scheduler for the framework proposed in Section 6.1. As shown, the application at the Customer Premises Equipment (CPE), i.e., the device transmitting with the different WATs, generates packets which are sent to the connection-level shared send buffer. Then, the scheduler decides which subflow queue to forward the next packet to based on the dynamically assigned weights.

### Setting WRR Weights

As previously mentioned, our implementation enables real-time modification of weights through *sysctl* system calls. This provides dynamic control over the proportion of traffic directed through each WAT, as explained earlier.

To maximize the aggregated throughput, our initial hypothesis is that this scheduler will enable us to achieve optimal throughput by setting the weights in proportion to the capacities of the different paths. Additionally, these weights will reduce packet reordering and waiting times at the receiver, thereby minimizing the HoL blocking problem and enhancing overall performance.

Therefore, to set the WRR weights, it is necessary to estimate the capacity of each WAT. Two approaches were devised for this purpose. The first approach involves directly measuring the capacity using network probes. In this method, a large number of packets are sent through each WAT without using MPTCP. The capacity is then estimated by dividing the total amount of traffic sent by the duration of the probe.

The second approach utilizes radio link and network Key Performance Indicators (KPIs) to estimate the capacity of each WAT through Machine Learning (or other AI approaches). This method, following solutions such as the one proposed in [30], employs a non-intrusive Machine Learning model to predict the available throughput.

In this work, we employed the first approach using the *iperf3* tool as it will be explained in Section 6.5. It is left for future work to estimate WATs’ capacities using the second approach.

## 6. User Plane Performance Evaluation

This section analyzes the end-user performance in a static scenario using multi-connectivity from three main points of view: throughput, latency, and WAT interruption recovery (time from which a technology is no longer used, e.g., due to lack of coverage, to when it continues to transmit as part of the MPTCP subflows). Additionally, throughput was also evaluated in a dynamic scenario.

### 6.1. Experimental Setup

The testbed used for this work was composed of the following WATs: 5GNR, Wi-Fi 6, and LiFi (configurations for our experiments are available at [31]).

For 5G, we employed one AMARI Callbox Classic from Amarisoft (which includes both the gNB and the core network) configured for 5G Stand Alone (SA) with MIMO 2 × 2 and 50 MHz of bandwidth. For our experiments, we employed two TDD patterns: one optimized for throughput (period 5 ms, seven DL slots, six DL symbols, four UL symbols, and two UL slots) and another optimized for latency (period 1 ms, one DL slot, zero DL symbols, twelve UL symbols, and zero UL slots). Due to the licensed band in which the system operated, we utilized a Faraday cage to enclose the gNB antennas and the device’s 5G modem. This ensured that no interference was transmitted or received from external sources, maintaining a controlled and isolated environment.

For Wi-Fi, our testbed included a Xiaomi AX9000 access point, which is IEEE 802.11ax-compliant, working with MU-MIMO 4x4 and a bandwidth of 160 MHz, allowing a peak data rate of 4804 Mbps. Using this bandwidth involved operating in the 5.2 GHz band, which minimized interference from nearby Wi-Fi access points. Additionally, since the experiments were conducted at the Higher Technical School of Computer and Telecommunication Engineering at the University of Granada, interference from residential Wi-Fi signals was minimal due to the school’s size and geographical location. However, to further reduce potential interference, including from the ETSIIT’s own access points, the experiments were carried out in the evening, when network traffic was lower and, consequently, interference was reduced.

Finally, the LiFi network contained a pureLiFi LiFi-XC access point and a LiFi-X USB dongle with a bandwidth of 16.6 MHz in the visible light spectrum (337 THz), which provided up to 42 Mbps. Since it uses light-frequency signals, the LiFi system is inherently immune to interference from other electromagnetic radiation. Consequently, no additional measures were needed in the testbed environment to prevent interference.

**Figure 3:**
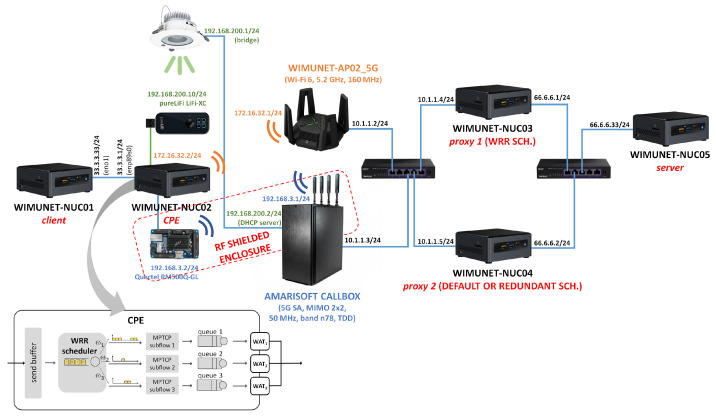
Multi-connectivity testbed.

The topology is shown in Figure 3. The five Intel NUCs had i7 processors with 16 GB of RAM and SDDs of 512 GB. The CPE (Customer Premises Equipment) included an Intel AX201 Wi-Fi 6 network card, a Quectel RM500Q-GL as a 5G modem, and the aforementioned LiFi-X USB dongle.

In the static scenario, the separation between the CPE and the Wi-Fi and LiFi access points was 1 and 2.5 m, respectively. The interconnection of the different pieces was performed with two 2.5 GbE switches. Since the CPE included three WATs and the proxy only used one network interface, three MPTCP subflows were established in the experiments.

### 6.2. Throughput Evaluation in a Static Scenario

The throughput evaluation was performed using the *iperf3* tool, which generated a sample every second. Throughput was measured in the downlink direction. The experiments lasted 10 min each and were performed at night to reduce interference from other Wi-Fi networks. In the experiments, 5G and LiFi did not receive interference from other networks due, in the first case, to the use of the RF shielded box and, in the second case, to its limited coverage under the luminaire. During this evaluation, the CPE location was fixed and placed next to the gNB and access points.

**Figure 4:**
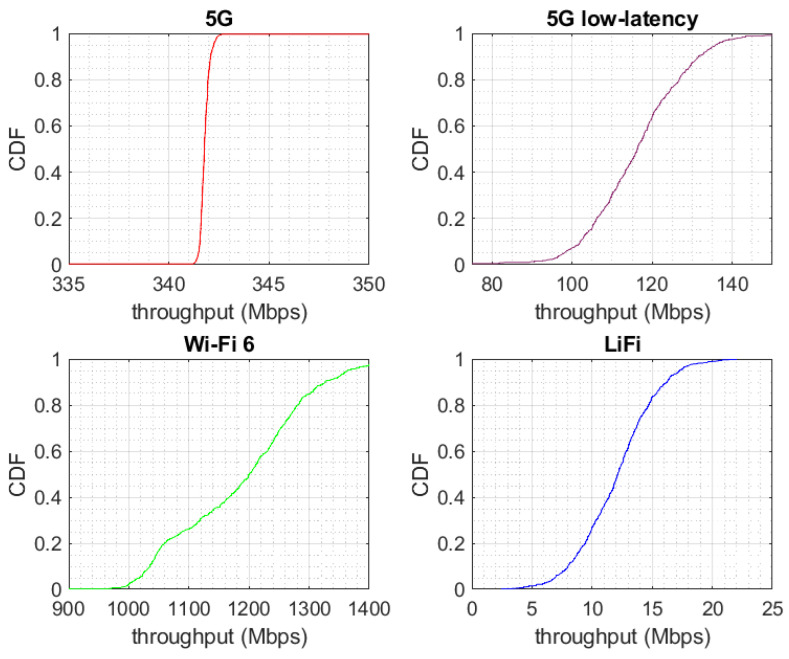
Throughput of different WATs.

**Figure 5:**
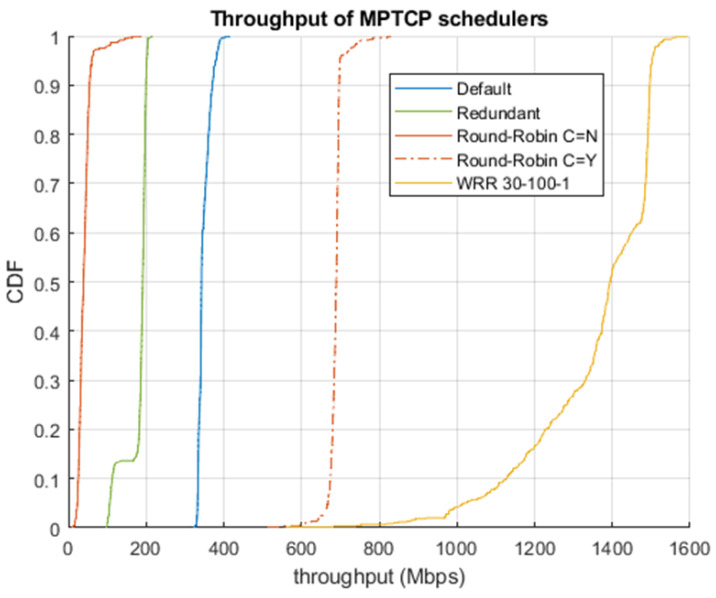
Throughput of different MPTCP schedulers.

The throughput performance of the different WATs and the different MPTCP schedulers is included in Figure 4 and Figure 5. Due to the use of a licensed band and isolation thanks to a Faraday cage, 5G was extremely stable on our testbed with an average throughput of 342 Mbps. In addition, the *5G configuration for low latency* was also included, since it could be tested on the latency evaluation subsection. This configuration achieved an average of 116 Mbps. Because of the usage of unlicensed frequencies and interference from nearby networks, Wi-Fi performance had some variability. Despite this, due to the proximity to the access point and the use of 160 MHz, Wi-Fi achieved a throughput between 1 and 1.4 Gbps, with an average of 1.19 Gbps. Finally, a high variability in the performance of LiFi was also appreciated, possibly as a result of the lack of technological maturity compared to the other WATs. Its throughput was between 5 and 20 Mbps, with an average value of 12.1 Mbps.

Regarding the performance of the MPTCP schedulers, the default and redundant schedulers are not designed to maximize throughput but to reduce latency and, in the case of the latter, improve reliability. For this reason, their throughput—which depends not only on the performance of each individual WAT but also on the temporal evolution of their congestion windows due to the HoL problem—was low in these experiments, achieving an average of 181 Mbps and 348 Mbps, respectively. In the case of the Round-Robin test, the performance strongly depends on the *CWND_LIMITED* parameter. If it is set to “*N*”, then a perfect Round-Robin policy is executed, so all interfaces transmit the same number of packets. This means that the slowest interface limits the rest. For that reason, the achieved throughput was very poor (41.1 Mbps on average, which is approx. 3 times the throughput of LiFi). If *CWND_LIMITED* is set to “*Y*”, the scheduler tries to fill the congestion window of each subflow, thus increasing the performance to approx. 690 Mbps.

However, using our WRR scheduler with the appropriate weights, we were able to achieve the maximum aggregated throughput. As commented in Section 5, we computed the weights to be approx. proportional to the data rate of each WAT: $\omega_{5G}=30$, $\omega_{WIFI}=100$, and $\omega_{LIFI}=1$, with 5G configured for maximum throughput, and $\omega_{5G}=10$, $\omega_{WIFI}=100$, and $\omega_{LIFI}=1$, with 5G configured for minimum latency. As shown, the throughput varies between 1 and 1.5 Gbps for the first case, with an average of 1.36 Gbps (roughly the sum of the averages of 5G, Wi-Fi, and LiFi and almost doubling that of RR), and around 200 Mbps less for the second case (due to the lower performance of *5G low latency*).

**Figure 6:**
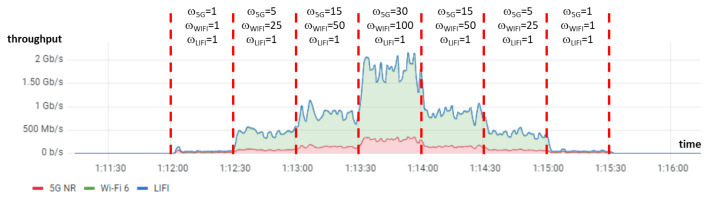
Aggregated throughput evolution depending on WRR weights.

Finally, to verify the correct functioning of our WRR scheduler, an experiment was carried out, increasing the weights every 30 s, from low values (1-1-1) to the values that maximize throughput (30-100-1). To accomplish this, we collected statistics with a customized script using the Socket Statistics (*ss*) tool, and the statistics were sent to Prometheus and shown with Grafana. The result is shown in Figure 6. Thanks to the weights, the percentage of traffic sent by each WAT can be distributed according to the capacity supported by each WAT, reaching the maximum aggregate if the weights are proportional to the capacities of those networks. It shall be noted that the curves in this figure are stacked and that the LiFi throughput is almost indistinguishable, as it is between one and two orders of magnitude lower.

### 6.3. Latency Evaluation

The latency evaluation was performed using the *netperf* tool (available at [32]), which was modified to collect the RTT for each packet. Other popular tools such as *ping* were not used since their packets are not transported over TCP and, thus, they are not suitable for MPTCP. Latency was measured at the CPE as one-way delay, computed as RTT/2, thus not requiring synchronization between the CPE and the MPTCP proxy. The experiments for latency evaluation were performed without considering other sources of traffic or interference. Other scenarios are left for further study. In addition, *tcpdump* (included in most Linux distributions and available at [33]) traces were captured to compute the percentage of packets sent via each WAT.

The experiments for each WAT consisted of a *netperf* run lasting 10 min. However, for the experiments with the different MPTCP schedulers, 100 repetitions were executed with a duration of 10 s and a pause of 1 s between repetitions. This ensures that the MPTCP behavior does not depend on a specific run of the experiment.

**Figure 7:**
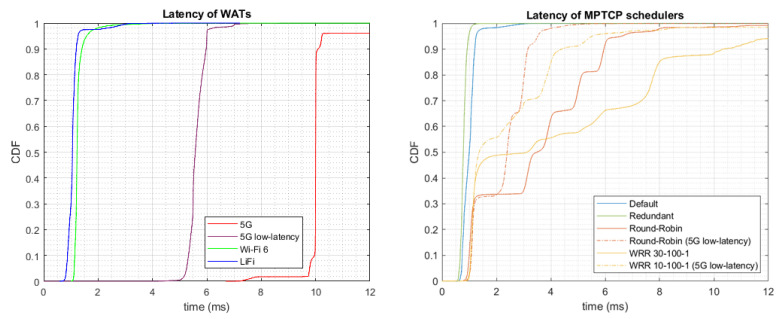
Latency (one-way delay) of (**left**) WATs and (**right**) MPTCP schedulers.

Figure 7 presents the latency of the different WATs and the different MPTCP schedulers. As shown, the one-way latency of LiFi and Wi-Fi was around 1 ms (1.08 ms and 1.28 ms on average, respectively), increasing to 10.1 and 5.6 ms for 5G and *5G low latency*, respectively.

As explained in Section 3, the MPTCP default scheduler selects the path with the lowest SRTT. Although SRTT depends on the instantaneous evolution of TCP, MPTCP mostly chooses LiFi and Wi-Fi due to lower latency compared to 5G. Thus, for the experiment carried out, LiFi sent 63.79% of the packets, Wi-Fi 35.93%, and 5G only 0.28%. The average latency and the 90th percentile were 1.00 and 1.20 ms, respectively.

With the redundant scheduler, packets are sent duplicated over all paths. Thus, the first packet received will be the one used by the application, so the latency will be similar to that of the interface with the lowest delay, LiFi or Wi-Fi, in this case. The average latency and the 90th percentile were 0.80 and 0.92 ms, respectively.

In the case of the RR or WRR, the latency is a mixture of the latencies of the different WATs. Using these MPTCP schedulers, a data packet and its corresponding ACK may be transmitted over different WATs. Thus, the RTT will be the sum of both latencies and the one-way delay, computed as RTT/2, will therefore be the average of the two WATs employed. Curves in Figure 7 include both 5G and *5G low latency* with the weights that maximize their aggregate throughput (weights 30-100-1 for 5G, Wi-Fi, and LiFi, respectively, and 10-100-1 for the case with *5G low latency*, as commented in Section 6.2). The average latency and the 90th percentile were 3.52 and 5.95 ms for RR, 2.25 and 3.15 ms for RR with *5G low latency*, 4.66 and 10.32 ms for WRR, and 2.55 and 4.38 ms for WRR with *5G low latency*, respectively.

### 6.4. WAT Interruption Recovery

The MPTCP approach for multi-WAT connectivity allows us to provide continuous transmission even when a link fails. This “always-on” type of connection replaces the need for a vertical handover across access networks.

In this subsection, we evaluate the time to resume the transmission when a new link becomes available, e.g., after crossing an area without coverage. It is noteworthy that the three WATs exhibit very different coverage footprints, which implies that intermittent coverage situations may arise, particularly for LiFi.

In order to check the availability of the interfaces, we used *NETLINK sockets* and *tcpdump* packet traces to find out when the transmission started again. No synchronization was required since both tools were executed on the CPE. The experiment considered 200 of these interruptions for each technology, which were forced by bringing down and up the corresponding network interface. This experiment was performed with a long MPTCP connection with 10 s transmitting, 5 s without transmitting through one of the WATs, and then resuming transmitting.

We split this time into two parts: a first part due to the association or registration in the network and a second from when the interface was available in the operating system until it was used again by MPTCP. The former depends on the particular network, equipment, and driver, while the latter only depends on the MPTCP implementation. The time to exchange DHCP messages should also be included if static IP addresses are not used.

**Figure 8:**
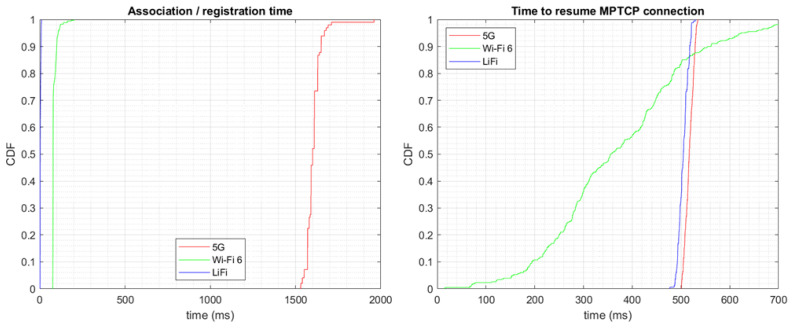
Time to recover after an interruption due to (**left**) association with/registration to the network and (**right**) MPTCP reconnection.

As shown in Figure 8, MPTCP reconnected after 0.1–0.7 s in the case of Wi-Fi, with an average of 393.3 ms. For 5G and LiFi, the interruption time was very stable with averages of 517.2 and 504.8 ms, respectively. We also tested with different Wi-Fi USB dongles, showing similar patterns with slightly higher interruption times. These values depend on the particular hardware, firmware, and device driver.

Regarding the time to associate with or register to the network, in the case of Wi-Fi and LiFi, it was measured from the moment the first *Authentication Request* was sent to when the *Association Response* was received. The scanning phase is not included in these results. As seen in Figure 8, this time was around 3–4 ms for LiFi (3.7 ms on average) and around 75 ms for most samples on Wi-Fi (83.9 ms on average). In the case of 5G, we measured the time between the first *RRC Setup Request* message and the last *RRC reconfiguration complete* after all the security negotiations and PDU session establishments. The average 5G registration time was around 1.5–1.7 s (1.60 s on average). It shall be noted that, due to the wide coverage of 5G compared to the other technologies, the loss of 5G connectivity is expected to be infrequent. Also, handovers are expected to take much less time as there will be no security negotiations. However, for the sake of completeness, we also included the time to register in a 5G network.

### 6.5. Throughput Evaluation in a Dynamic Scenario

The objective of this experiment is to check whether the WRR weights can be adjusted in real time depending on the radio link conditions in order to achieve the maximum possible throughput. As an initial hypothesis, as commented in Section 6.2, we assume that maximum aggregate throughput is achieved when the weights are proportional to the capacities of the different WATs.

In a realistic indoor environment with a private 5G network (see Section 3.4.2.1 in [34]), 5G performance degrades slowly due to the low co-channel interferences since 5G bands are licensed. However, Wi-Fi performance decreases rapidly with distance.

**Figure 9:**
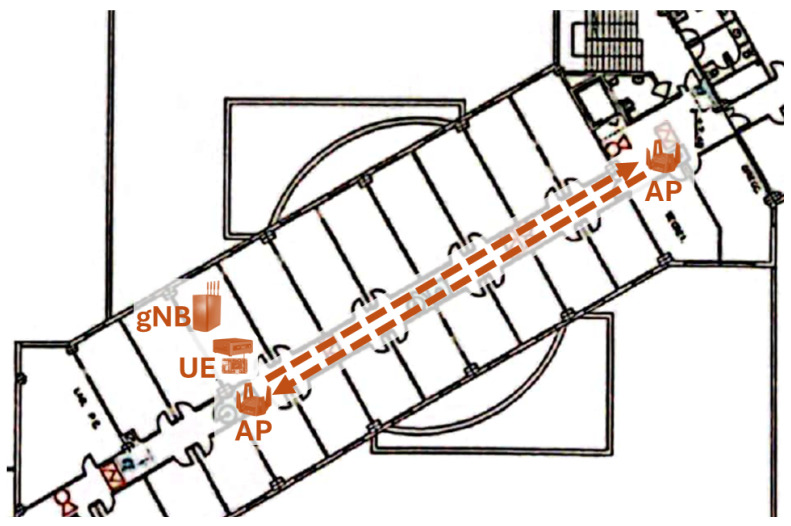
Dynamic scenario emulating the movement of a mobile device.

For these reasons, we chose to conduct an experiment in which the mobile device was placed near the gNB (which is an indoor unit for laboratories) and the Wi-Fi access point was moved away, attempting to emulate the indicated radio conditions. Since the mobile device was assumed to be moving during the experiment, LiFi was not considered. Figure 9 shows the layout of the location where this experiment was conducted (at the Higher Technical School of Computer and Telecommunication Engineering of the University of Granada), including the movement of the Wi-Fi access point. The length of the round trip route was approximately 50 m, which was covered at a steady pace in 4 min and 10 s.

**Figure 10:**
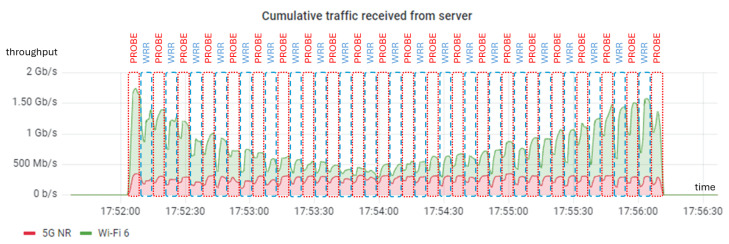
Aggregated throughput in the dynamic experiment.

**Figure 11:**
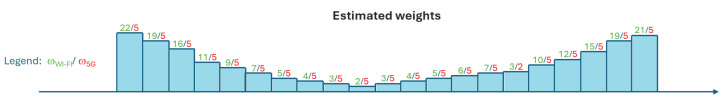
Weights estimated using probes in the dynamic experiment.

In order to obtain the capacity of the different WATs, we first executed periodic probes every 10 s using the *iperf3* tool with a duration of 5 s and 10 parallel flows. These probes used normal TCP to avoid being affected by MPTCP. It is left for future work to estimate these capacities based on radio or network KPIs using methods such as Machine Learning. As an initial validation, we conducted another *iperf* connection of 5 s using MPTCP with the WRR scheduler after each probe. As shown in Figure 10, the throughput achieved with WRR and these weights (in the blue discontinuous boxes with labels “WRR”) closely matched the throughput measured during the probes (in the red discontinuous boxes with labels “PROBE”). Figure 11 includes the weights estimated during the probes—proportional to the estimated WAT capacities—which will be used in the next experiment. The height of each step is proportional to the ratio between the weights for Wi-Fi and 5G. For simplicity, capacities were rounded to the nearest 10 Mbps when estimating weights.

**Figure 12:**
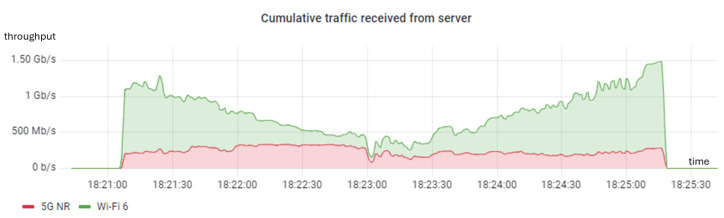
Dynamic experiment using MPTCP and the WRR scheduler.

Following the weight estimation, we conducted an experiment in the same environment using a continuous *iperf* connection with MPTCP and WRR. The weights used were identical to those used in the previous experiment and were updated every 12 s (a total of 22 times throughout the entire route). Figure 12 illustrates the cumulative throughput, which aligns precisely with the capacity estimated earlier (see Figure 10). It is important to highlight that, although we tried to replicate the conditions of the previous experiment as closely as possible, identical radio link conditions could not be guaranteed due to interferences from other Wi-Fi access points, reflections from walls, fading, and other factors. Nonetheless, these results indicate that (1) weights can be dynamically adjusted in real time to adapt to changing radio link conditions and (2) the aggregated throughput is optimized when the weights are proportional to the capacities of the different WATs.

## 7. Conclusions and Future Work

In this paper, we analyzed the performance of some relevant Multi-Path TCP schedulers using a real testbed with three Wireless Access Technologies: 5GNR, Wi-Fi, and LiFi. To the best of our knowledge, this is the first work in the literature to consider multi-connectivity across 5GNR, Wi-Fi, and LiFi. In addition, we developed an MPTCP Weighted Round-Robin scheduler which allows a mobile operator to (1) maximize the aggregated throughput and (2) control the proportion of traffic sent through each WAT.

We evaluated three different aspects: throughput, latency, and recovery after coverage loss. Regarding throughput, our MPTCP WRR scheduler was able to achieve the sum of the maximum capacity of the different technologies, around 1.4 Gbps in our tests, far outperforming the other tested MPTCP schedulers (default, redundant, and Round-Robin).

In terms of latency, both the redundant and the default schedulers achieved the performance of the technology with lower delay, i.e., around 1 ms thanks to Wi-Fi and LiFi. All MPTCP schedulers are intrinsically reliable due to the usage of TCP.

Finally, MPTCP provides continuous transmission even when a link fails, so there is no real vertical handover when using multi-connectivity. We measured the time to recover after an interruption, e.g., due to lack of coverage in one technology, which was around 400-500 ms (in addition to the time to register to/associate with the network, if needed).

Additionally, we evaluated the performance in a dynamic scenario to assess the proper behavior of our scheduler under varying conditions. As demonstrated, when weights are proportional to the capacities of the different WATs, the aggregated throughput is approximately equal to the sum of those capacities.

Although this work focuses on performance within a laboratory testbed, it can be extended to other environments. As mentioned, this multi-connectivity framework was validated in a real production environment provided by BOSCH in Aranjuez (Madrid, Spain) [10], utilizing the MPTCP Round-Robin scheduler for capacity aggregation services, and the MPTCP redundant scheduler for latency-sensitive services. Other sectors that could benefit from 5G multi-connectivity to support breakthrough services include tourism, healthcare, retail, transportation hubs, sports facilities, and manufacturing, among others. These sectors will require diverse, complex, and stringent wireless access demands, making them well suited for the integration of 5G, Wi-Fi, and LiFi technologies.

Left for future work is the implementation of an Artificial Intelligence (AI)-based xAPP within the near-Real-Time RAN Intelligent Controller (near-RT RIC) for the online calculation of the weights based on the KPIs of the different technologies, e.g., using an Open-Source framework such as Open AI Cellular [35]. This way, aggregate throughput will be automatically optimized under changing radio link conditions. One key challenge with this approach is that the measurements are typically aggregated at the cell level, which means they do not accurately represent the performance of individual connections. Additionally, unlike cell capacity metrics, user capacity may not have a direct linear relationship with cell-level indicators [25]. Another challenge lies in integrating these frameworks into the 5G laboratory network, particularly at the database and API levels.

In addition, we plan to design, implement, and evaluate new dynamic packet schedulers that leverage hybrid online and offline learning approaches, using both MPTCP and MPQUIC (Multi-Path Quick UDP Internet Connection) to improve radio resource utilization and optimize KPIs such as cumulative throughput, end-to-end latency, and reliability during real-time packet delivery. For this purpose, we intend to employ deep reinforced learning (DRL) algorithms such as Deep Q-Network, Asynchronous Advantage Actor-Critic (A3C), or Proximal Policy Optimization (PPO). These implementations can be performed at the kernel level as an embeddable module or at the user plane level as a framework that observes the state of the radio interfaces and updates the weights of the scheduler proposed in this work in real time.

## Data Availability

The original contributions presented in the study are included in the article, further inquiries can be directed to the corresponding author.
